# Integrated process for lignin depolymerization and nanoparticle production using deep eutectic solvent

**DOI:** 10.1038/s41598-025-96237-7

**Published:** 2025-04-06

**Authors:** Jungkyu Kim, Chaeeun Kim, Chaewoo Jeong, Sungwook Won, Seon-Gyeong Kim, Hyoseung Lim, Seojin Kim, Hyo Won Kwak

**Affiliations:** 1 Department of Agriculture, Forestry and Bioresources, College of Agriculture & Life Sciences, Seoul National University, 1 Gwanak-ro, Gwanak-gu, Seoul, 08826 Republic of Korea; 2 Research Institute of Agriculture and Life Sciences, Seoul National University, 1 Gwanak-ro, Gwanak-gu, Seoul, 08826 Korea

**Keywords:** Nanoscale materials, Materials science, Biomaterials

## Abstract

Lignin, the most abundant natural aromatic macromolecule, holds significant potential for high-value applications. However, its complex and irregular structure, along with challenges in efficient processing, has limited its widespread use. In this study, we propose an ecofriendly continuous process utilizing deep eutectic solvents (DESs) for lignin depolymerization and subsequent production of lignin nanoparticles. DESs, composed of choline chloride and lactic acid, effectively break down lignin into low-molecular-weight homogeneous fragments while also serving as a solvent for nanoparticle formation. The depolymerized lignin nanoparticles (DLNPs) exhibited a uniform particle size distribution and enhanced surface charge due to interactions with the DES, resulting in excellent long-term dispersion stability. Chemical analyses indicated that lignin depolymerization primarily involved the cleavage of β-O-4 linkages while retaining its aromatic structure and forming condensation products such as pinoresinol and phenylcoumaran. Thermal analysis revealed that DLNP produced through this continuous process displayed improved thermal stability compared to kraft lignin, suggesting potential applications in high-temperature environments, such as flame retardants. This study demonstrates that the DES-based process is a promising alternative to conventional organic solvent methods, providing a sustainable and efficient pathway for lignin nanoparticle production and valorization.

## Introduction

Lignin is one of the most abundant natural aromatic biopolymers, holding great potential for conversion into functional materials due to its three-dimensional network structure of phenylpropane units and its rich array of functional groups^1,2^. Current research extensively explores lignin’s applications in energy, particularly in batteries and supercapacitors, as well as in environmental remediation materials such as dye and heavy metal adsorbents^3–8^. Recently, with growing interest in nanotechnology, attention has increasingly focused on the application of lignin nanoparticles (LNPs). Owing to their large surface area and excellent dispersibility in various liquids, LNPs have garnered considerable attention in biotechnology and chemical engineering for applications in bioactive composite materials and Pickering emulsions^9–12^.

To successfully manufacture LNPs and achieve their effective high-value utilization, it is essential to address the complex composition and structural heterogeneity of lignin. Traditional methods often rely on fractionation processes using various solvents. Fractionation allows for control over the molecular weight and chemical structure of lignin, enhancing its homogeneity and enabling the production of lignin suitable for high-value applications^13–15^. Common solvents used in lignin fractionation include acids, alkaline solvents, acetone, dimethyl sulfoxide (DMSO), dioxane, hexane, tetrahydrofuran (THF), and methanol^16–20^. However, these organic solvents are highly volatile and toxic, leading to environmental issues such as air and water pollution, and posing serious health risks from human exposure^21,22^. In addition, highly flammable organic solvents raises concerns about fire and explosion hazards during processing. Consequently, there is an increasing demand for environmentally friendly lignin purification methods to replace conventional fractionation processes.

Lignin depolymerization using deep eutectic solvents (DESs) is a method that improves the limitations of organic solvent–based fractionation processes while ensuring the homogeneity of the resulting lignin. DESs, comprising hydrogen bond acceptors (HBAs) and hydrogen bond donors (HBDs), have attracted attention for lignin depolymerization owing to its low-cost raw materials, low toxicity, excellent solubility, and recyclability, positioning it as a more ecofriendly alternative to conventional solvents^23–25^. DES facilitates the cleavage of unstable ether bonds between phenylpropane units in lignin through acid–base catalysis, resulting in low-molecular-weight homogeneous lignin^26^. Among these, acidic DES using choline chloride (ChCl) as an HBA is extensively studied for lignin depolymerization due to its effective lignin solubilization capacity, mild reaction conditions, and non-volatility^27,28^. Additionally, lactic acid (LA) as an HBD in acidic DES enhances the number of active protons and improves proton mobility in the ChCl/LA DES, thereby fostering better interactions between lignin and the solvent and promoting the breakdown of ester bonds in lignin^29–31^.

DES can also serve as an ecofriendly solvent in the production of LNPs using the antisolvent method, replacing organic solvents such as acetone, DMSO, and THF^32–35^. Luo et al.^36^ employed binary DES, with ChCl as the HBA and various HBDs, to produce LNPs under different lignin types, HBDs, and concentration conditions. Their findings revealed that LNPs with adjustable diameters could be consistently produced by controlling process variables. Similarly, Zheng et al.^37^ used DES composed of ChCl and EG to produce LNP solutions at varying concentrations. They also incorporated the LNPs into PVA to fabricate PVA/LNP nanocomposite films, which exhibited excellent antioxidant and antimicrobial properties. These studies demonstrate that LNPs can be produced using DES under diverse conditions, with size and shape controlled through process adjustments. Moreover, the dissolution process using DES preserves the inherent properties of lignin, allowing DES to fully replace organic solvents in LNP production.

This study presents an eco-friendly and energy-efficient approach by integrating DES-based lignin depolymerization with LNP production into a single streamlined process. Unlike conventional methods that separately conduct lignin fractionation or depolymerization before nanoparticle fabrication—leading to excessive energy and solvent consumption—this integrated strategy optimizes resource utilization. Kraft lignin (KL) was depolymerized with DES, using ChCl as the HBA and LA as the HBD. The DES also functioned as a solvent in the ecofriendly LNP production process, utilizing water as an antisolvent. Gel permeation chromatography (GPC) was employed to identify optimal depolymerization conditions for the most homogeneous lignin. Additionally, the morphological characteristics and dispersion stability of the depolymerized lignin nanoparticles (DLNPs) obtained from the subsequent LNP production process were evaluated. Fourier-transform infrared spectroscopy (FTIR), quantitative 31-phosphorous nuclear magnetic resonance (^31^P-NMR), X-ray photoelectron spectroscopy (XPS), and two-dimensional heteronuclear single quantum coherence (2D–HSQC) NMR spectroscopy were used to specifically assess the chemical changes in lignin resulting from depolymerization and condensation. Furthermore, the thermal properties of lignin, modified during the depolymerization process, were also examined. The controlled condensation occurring during the DES-based process enhances the thermal properties of the final LNP product, further improving its performance and stability. By demonstrating the feasibility of this approach, our study highlights the potential for sustainable and scalable applications of LNPs in various fields.

## Materials and methods

### Materials

KL was generously obtained from Moorim P&P (Ulsan, Korea). ChCl (99.0%) and LA (95.0%) were purchased from DUKSAN Science (Seoul, Korea). THF (99.9%) was purchased from SAMCHUN Chemicals (Pyeongtaek, Korea). CDCl_3_, anhydrous pyridine, chromium acetylacetonate (III), cyclohexane DMSO-*d*_*6*_, and 2-chloro-4, 4, 5, 5-tetramethyl-1, 3, 2-dioxaphosphane (TMDP) were purchased from Sigma-Aldrich Korea (Seoul, Korea).

### Preparation of DES

DES was prepared by mixing ChCl and LA in a 1:1 molar ratio, followed by heating and stirring in a 60 °C-oil bath until a homogeneous and transparent liquid was achieved. The prepared DES was then sealed and stored in a desiccator to prevent moisture absorption before use.

### Integrated process of lignin depolymerization and nanoparticle production using DES

**Fig. 1:**
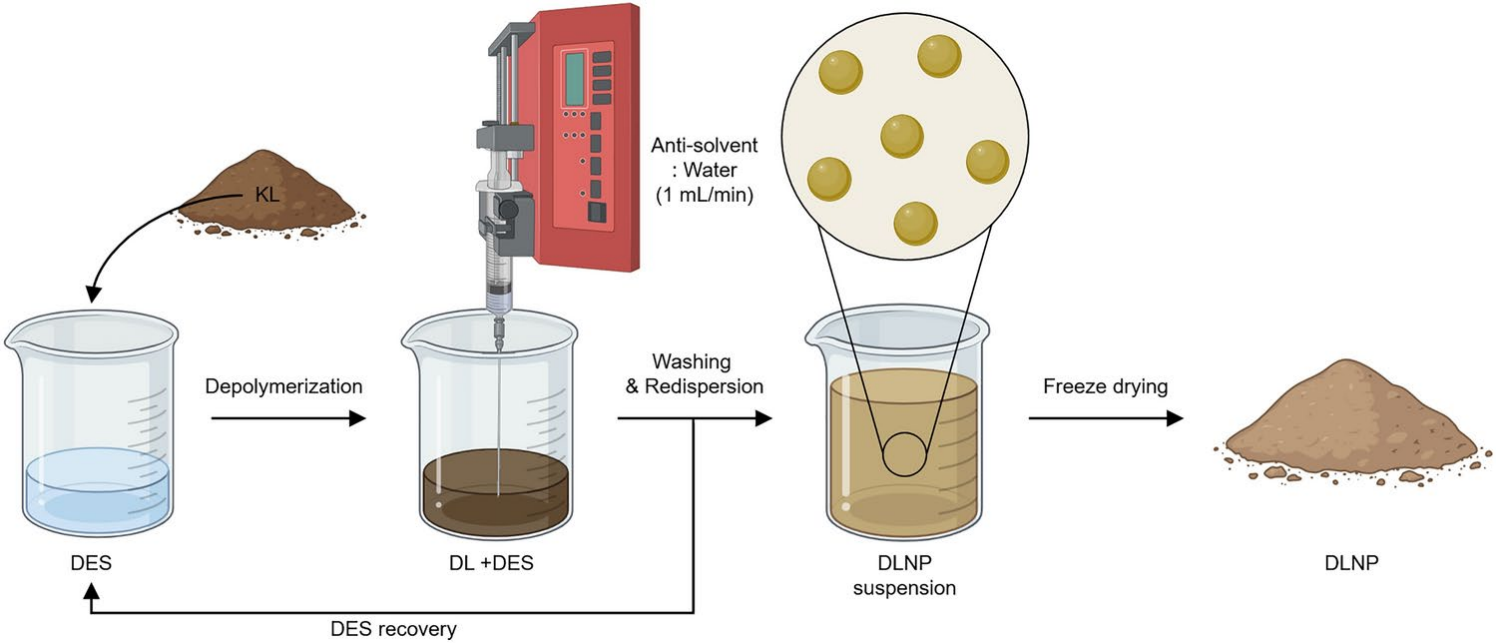
Schematic illustration of the depolymerization of KL and the subsequent production of nanoparticles using DES.

The overall process for lignin depolymerization and LNP production is illustrated in Fig. 1. For lignin dissolution and depolymerization, 1 g of KL and 20 g of DES were combined in a 200-mL flask, with the reaction conducted at various temperatures (60–180 °C) and times (2–24 h) using an oil bath. To terminate the reaction, the flask containing the lignin solution was cooled in an ice chamber. Lignin nanoprecipitation was induced by stirring the lignin solution at 500 rpm at room temperature while adding 50 mL of distilled water (DW) as an antisolvent at a rate of 0.5 mL/min using a syringe pump (NE-1000, New Era Pump Systems, New York, USA). To remove the DES and soluble lignin depolymerization products, the solution was centrifuged at 15,000 rpm for 2 min, and the supernatant was discarded. This washing and centrifugation step was repeated twice to recover clean DLNPs.

Depolymerized lignin (DL) refers to lignin that has undergone depolymerization but omits the nanoparticle fabrication process. DL is produced through a process similar to that of DLNP. After depolymerization, 50 mL of DW is added to the lignin-DES solution, followed by centrifugation and DW washing to obtain DL. The weight of the obtained DLNPs was measured to calculate the yield of the DL. Finally, the DLNPs were frozen at − 65 °C and freeze dried.

### Characterization of KL and DLNPs

The purity of lignin was determined based on the quantification of Klason lignin and acid-soluble lignin following the NREL laboratory analytical procedure^38^. The absorbance at 205 nm for measuring acid-soluble lignin contents was determined using a UV–Vis spectrometer (OPTIZEN POP, KLAB, Korea). The molecular weight of KL and DLNP was assessed using a NEXERA (Shimadzu, Kyoto, Japan). Each sample was dissolved in HPLC-grade THF and filtered through a 0.45-micron polytetrafluoroethylene syringe filter (Advantech Co., Tokyo, Japan). Molecular weight measurements were conducted at 40 °C with a flow rate of 0.1 mL/min. The refractive index detector used was the RID-20A from Shimadzu Corporation (Kyoto, Japan), and the column was the PSS SDV analytical 1000 Å from PSS (Mainz, Germany). Prior to measurements, polymethyl methacrylate and polystyrene were employed as standard materials.

Optical images of KL, DL, and DLNPs were captured using a Galaxy S22 digital camera (Samsung, Suwon, Korea). Field emission scanning electron microscope (FE-SEM) images of KL and DLNP were obtained with a SIGMA microscope (Carl Zeiss, Jena, Germany) operating in secondary electron mode at an acceleration voltage of 5 kV. Prior to FE-SEM analysis, the samples were coated with a 10-nm-thick platinum layer using an EM SCD005 sputtering coater (Leica, Germany). The morphology of DLNP was examined using transmission electron microscopy (TEM, JEM2010PLUS, JEOL, Tokyo, Japan) at an acceleration voltage of 80 kV. A 0.01-wt% dispersion of DLNPs was prepared, and a single drop was cast on a carbon-coated Cu grid (200-mesh size). The diameter of the DLNPs was calculated using the ImageJ program (National Institutes of Health, Maryland, USA).

The Litesizer 500 (Anton-Paar, Graz, Austria) was used to measure the zeta potential and hydrodynamic diameter of the DLNPs. The zeta potential was analyzed at pH 7, 25 °C, with an adjusted voltage of 200 V and 1000 scans. Dynamic light scattering (DLS) tests were also conducted under the same conditions (pH 7, 25 °C). The zeta potential and hydrodynamic diameter of the DLNPs were measured three times, and the average values were reported.

FTIR analysis was conducted to characterize the functional groups of lignin using the attenuated total reflectance (ATR) mode. FTIR-ATR spectra were recorded in the range of 4000–500/cm, with 32 scans and a resolution of 4/cm, using a Summit instrument (ThermoScientific Inc., Massachusetts, USA).

The hydroxyl groups of KL and DLNP were quantified using 31P-NMR analysis with an Advance III HD 600 MHz high resolution NMR spectrometer (Bruker, Germany). To prepare lignin for 31P-NMR analysis, 40 mg of KL and DLNP was phosphitylated using TMDP. The dried lignin was dissolved in 500 μL of anhydrous pyridine/CDCl_3_ solution (1.6:1, v/v) containing 50 μL of acetylacetonate chromium (III) as a relaxation reagent.

The chemical structure of KL and DLNPs was further characterized using the XPS and 2D–HSQC NMR technique. The chemical structure of KL and DLNPs was further characterized using the XPS and 2D–HSQC NMR techniques. XPS analysis was conducted using an AXIS SUPRA X-ray photoelectron spectrometer (Kratos, UK) within the binding energy range of 0 eV to 1200 eV. The C/O ratio was determined by integrating the XPS spectra area corresponding to the binding energy ranges of C 1 s and O 1 s. Each peak was deconvoluted using a Gaussian function. 2D–HSQC NMR performed on an Advance III HD instrument. A 40-mg lignin sample was dissolved in 750 μL of DMSO-*d*_*6*_ at 25 °C. ^13^C NMR and ^1^H NMR were measured in the ranges of 45–140 ppm and 2.5–8.0 ppm, respectively.

Thermogravimetric analysis (TGA) of lignin before and after depolymerization was conducted using a Discovery TA instrument (TA instruments, Delaware, USA). A 10-mg lignin sample was heated from room temperature to 800 °C at a rate of 10 °C/min under a nitrogen gas flow of 30 mL/min, generating TGA and derivative thermogravimetry (DTG) curves. Differential scanning calorimetry (DSC) thermograms of KL and DLNPs were recorded using an SDT Q600 (TA instruments, Delaware, USA). DSC measurements were performed under a nitrogen purge at a flow rate of 30 mL/min, with a heating rate of 10 °C/min over a temperature range of 30–300 °C.

## Results and discussion

The results of the molecular weight analysis conducted to optimize the lignin depolymerization conditions using DES are presented in Table 1. Analysis of the molecular weight of DL at varying reaction temperatures showed that almost no depolymerization occurred at 60 °C. As the temperature increased, the number-average molecular weight (M_n_), weight-average molecular weight (M_w_), and PDI of the DL consistently decreased, indicating that DES effectively cleaved the β-O-4 bonds in lignin, resulting in more homogeneous lignin fragments. Additionally, the increase in purity and decrease in yield result from the simultaneous effects of residual carbohydrate removal in KL and the loss of low-molecular-weight depolymerization products generated during depolymerization. Given that thermal decomposition of lignin occurs at temperatures above 200 °C, the optimal temperature for lignin depolymerization was determined to be 180 °C^39^. Increasing the reaction time also led to a decrease in M_n_ and M_w_. However, when the reaction time exceeded 12 h, the yield of DL dramatically declined, so optimal reaction time was determined to be 7 h. Notably, extending the reaction time to 24 h resulted in higher molecular weight and PDI compared to those of lignin depolymerized for 12 h. This phenomenon was attributed to a crossover in which the condensation, occurring simultaneously with depolymerization, became dominant as the reaction time increased. Under the optimal conditions of 180 °C for 7 h, the Mw of lignin was reduced by 40.8%, and the PDI decreased from 3.37 to 2.31. These results confirm that the DES-based depolymerization process produced lignin with a lower and more homogeneous molecular weight, demonstrating its potential as an eco-friendly alternative to conventional organic solvent-based lignin fractionation methods.

**Table 1:** GPC results, purity, and yield of KL and DL as a function of depolymerization temperature and time.

	M_n_	M_w_	PDI	Purity (%)	Yield (%)
KL	718	2423	3.37	86.3	–
DL_60°C_7h	727	2580	3.55	90.9	98.3
DL_100°C_7h	697	2295	3.30	91.4	80.5
DL_140°C_7h	603	1901	3.16	92.4	75.3
DL_180°C_2h	605	1857	3.07	88.5	67.9
DL_180°C_7h	619	1435	2.31	95.1	69.3
DL_180°C_12h	514	1430	2.78	95.0	30.6
DL_180°C_24h	530	1541	2.93	95.9	30.4

**Fig. 2:**
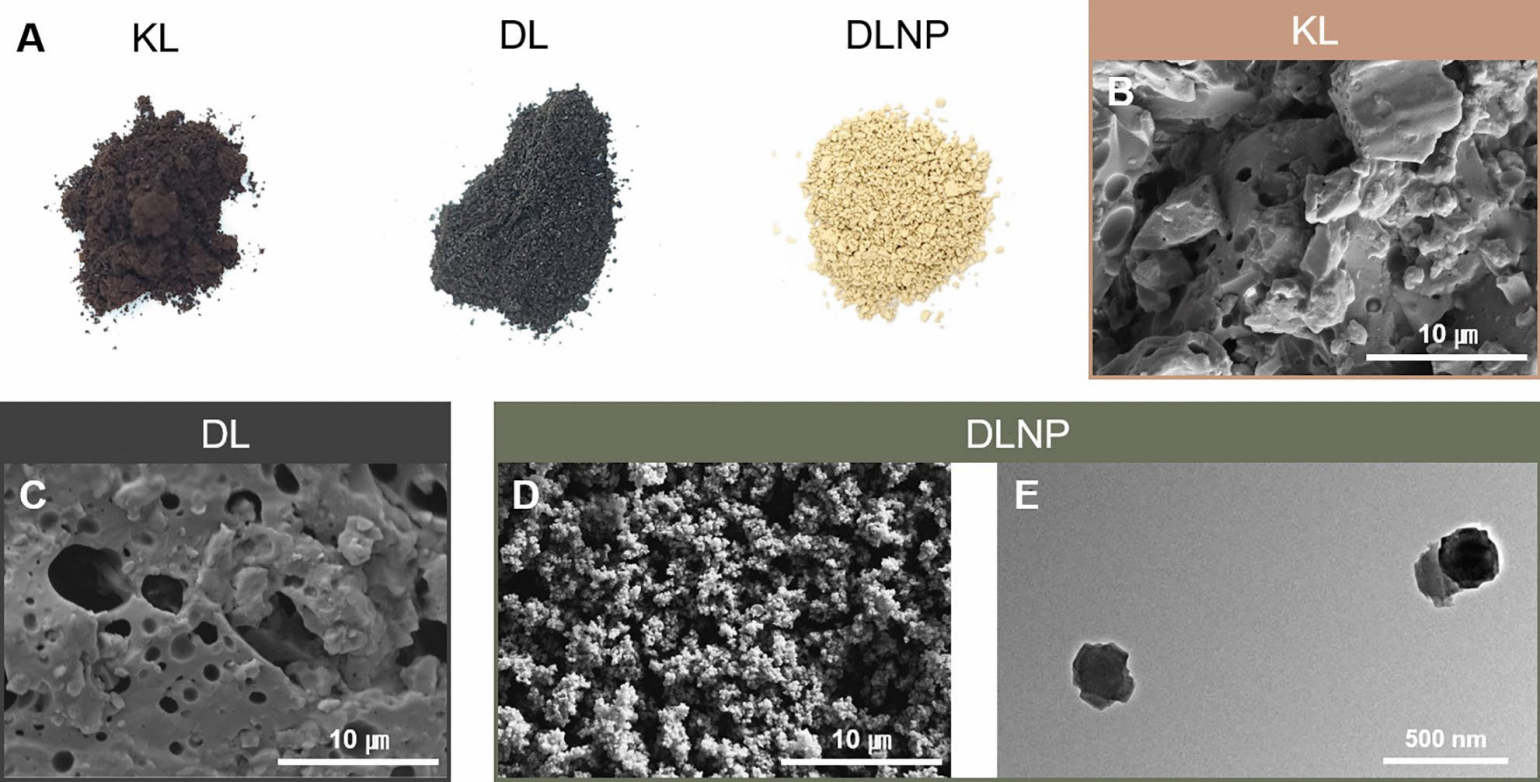
(**A**) Optical images and (**B**–**D**) FE-SEM images of KL, DL, and DLNP. (**D**) TEM images of DLNP.

The production process of LNPs can be continuously applied following the lignin depolymerization process using DES. DES functions not only as a solvent for lignin depolymerization but also as a solvent for nanoparticle production, enabling efficient LNP production directly from the mixture of DL and DES. The morphological characteristics of KL, DL and DLNP are depicted in Fig. 2. In Fig. 2A, KL retains its characteristic yellowish-brown color, while DL appears black, a color change attributed to the condensation structures formed during depolymerization^40^. Conversely, the conversion of DL into DLNP results in a lighter yellow color, likely due to the lower particle density of DLNP^41^. The scattering of light through the numerous spaces between particles contributes to this lighter color compared to DL. These observations are further supported by the FE-SEM images in Fig. 2B–D. While KL exhibits a smooth microscale particle morphology, numerous micropores are observed on the surface of DL after DES depolymerization. These micropores are likely traces of gas release that occurred during the decomposition of lignin in the DES treatment process. Additionally, DLNP appears as aggregates composed of LNP particles with sizes ranging from 100 to 200 nm. These LNP aggregates are loosely formed, enhancing the brightness of DLNP compared to KL and DL. To examine the individual particle morphology and size of DLNP within the aggregates, TEM analysis was conducted after preparing a DLNP suspension, as shown in Fig. 2D. Consistent with the FE-SEM findings, DLNP exhibits a wrinkled shape with a rough surface and a diameter of approximately 180–200 nm.

**Fig. 3:**
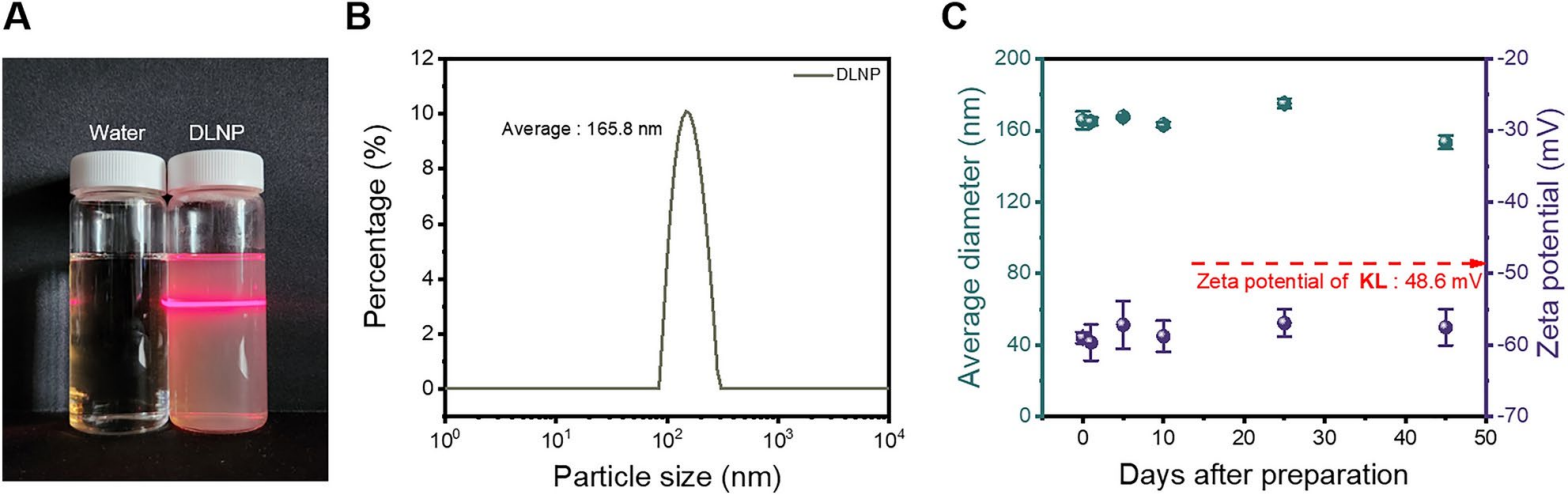
(**A**) Image demonstrating the Tyndall effect in DLNP. (**B**) Particle size distribution of DLNP. (**C**) Average diameter and zeta potential of DLNP as a function of storage time.

The hydrodynamic diameter and zeta potential of DLNPs were measured, as shown in Fig. 3. The DLNP suspension clearly exhibits the Tyndall effect, illustrated in Fig. 3A, which occurs when light is scattered by particles uniformly dispersed in a suspension within the size range of 1–1000 nm^42^. The DLS measurement in Fig. 3B confirms that the DLNPs, with an average hydrodynamic diameter of 165.8 nm, are well dispersed, further demonstrating the Tyndall effect. This hydrodynamic diameter aligns with the diameter observed in Fig. 2. Zeta potential, an important indicator of surface charge, plays a crucial role in understanding electrostatic interactions within colloidal systems^43^. As shown in Fig. 3C, the initial zeta potential of DLNP was − 58.9 mV, which was higher than that of KL (− 48.6 mV). The high zeta potential of DLNP can be attributed to the exposure of polar functional groups, such as phenolic hydroxyl and methoxy groups, on the lignin surface during the LNP preparation process using DES and water. DLNP maintained a zeta potential of higher than − 57.5 mV for 45 days, which contributed to its excellent dispersion stability without self-aggregation over this period. Further analysis of the reaction between lignin and DES is discussed in the following section.

**Fig. 4:**
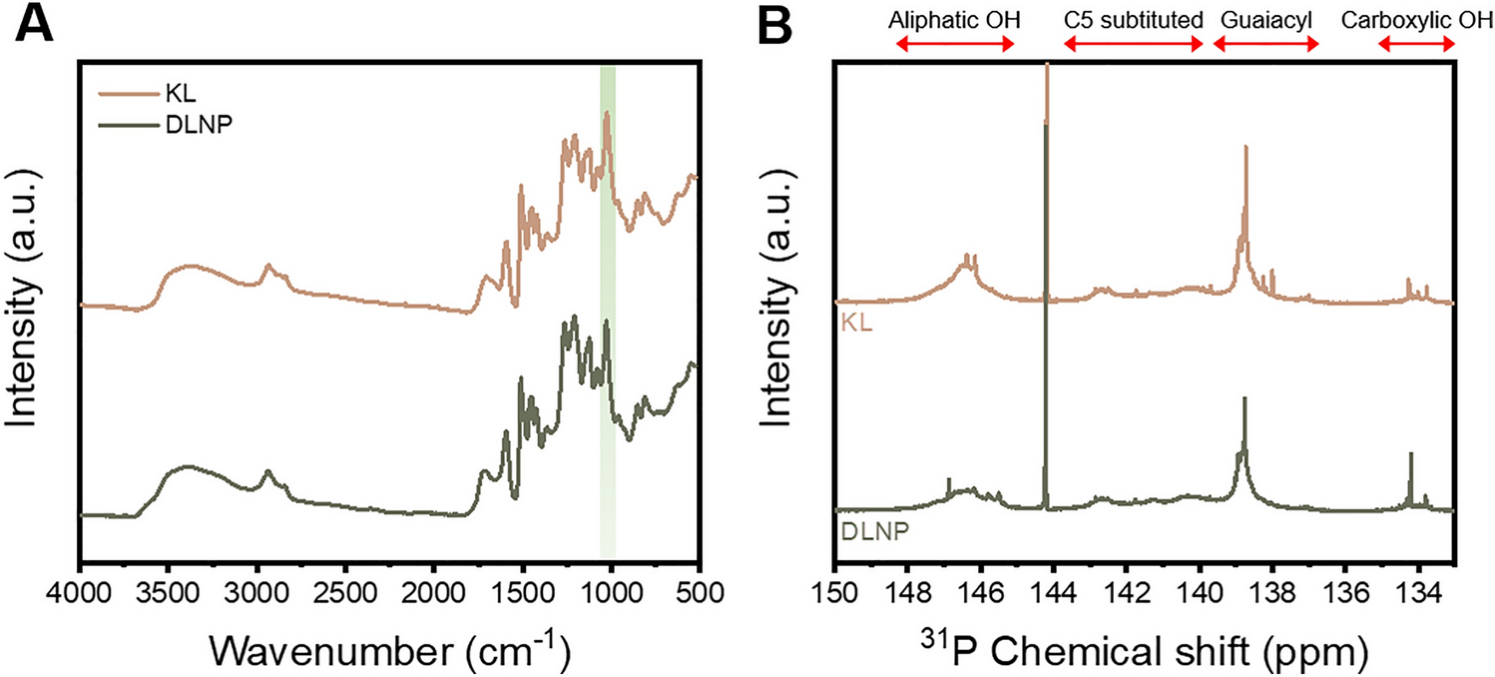
(**A**) FTIR and (**B**) ^31^P NMR spectra of KL and DLNP.

The FTIR spectra comparing the chemical structures of KL and DLNPs are shown in Fig. 4A. KL exhibited a broad OH stretching peak at 3600–3200/cm, alkyl stretching peaks at 2934 and 2845/cm, a carbonyl stretching peak at 1720/cm, and peaks associated with the lignin aromatic skeleton at 1592, 1514, and 1450/cm^44^. Additionally, characteristic signals of guaiacyl units were observed at 1126 and 1365/cm, syringyl units at 1205 and 1270/cm, and p-hydroxyphenyl units at 857 and 808/cm, representing a typical spectrum of lignin derived from woody biomass^45–47^. The FTIR spectra of DLNP were generally similar to those of KL, indicating that the DES-based depolymerization process did not have a destructive impact on the internal structure of lignin. However, a reduction in the peak at 1030/cm, corresponding to ether bonds, was noted in the DLNP spectra, suggesting the cleavage of the β-O-4 bond during depolymerization.

Lignin can achieve high-value-added applications by imparting special properties through various modification methods. Most modifications target aliphatic and phenolic hydroxyl groups, making the preservation of hydroxyl groups during depolymerization crucial. Changes in lignin hydroxyl groups due to depolymerization using DES were quantified via ^31^P-NMR, as shown in Fig. 4B and Table 2. After depolymerization, aliphatic OH and phenolic OH decreased by 0.73 mmol/g and 0.26 mmol/g, respectively. The reduction in aliphatic OH was 35.6%, which exceeded the reduction in phenolic OH, likely due to radical formation starting at the Cα OH during the depolymerization process^48^. Overall, 82.4% of the hydroxyl groups were preserved, demonstrating the potential of DLNP for various applications through subsequent modification processes.

**Table 2:** Quantitative ^31^P-NMR results for KL and DLNP.

	Aliphatic OH (mmol/g)	Phenolic OH(mmol/g)			Carboxylic group (mmol/g)	Total OH (mmol/g)
		C5 substituted	Guaiacyl	Total phenolic OH		
KL	2.05	1.39	1.84	3.23	0.12	5.391
DLNP	1.32	1.36	1.61	2.97	0.15	4.444

**Fig. 5:**
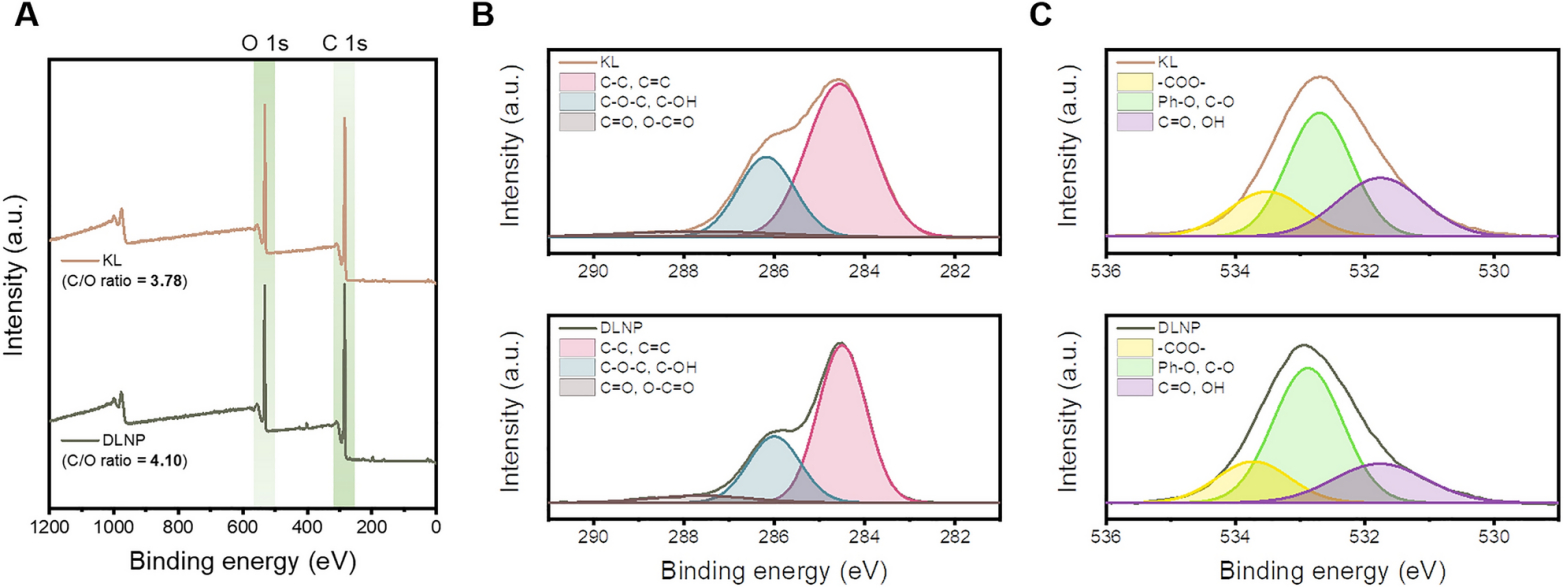
(**A**) XPS total survey, (**B**) C 1s, and (**C**) O 1s spectra of KL and DLNP.

The chemical bond changes in lignin before and after depolymerization using DES were analyzed by XPS, and the results are presented in Fig. 5. In the full scan shown in Fig. 5A, both KL and DLNP exhibited peaks corresponding to C 1s and O 1s at approximately 285 eV and 533 eV, respectively. Compared to the XPS spectra of KL, the O 1s peak of DLNP showed a decrease in intensity, and the C/O ratio increased from 3.78 to 4.10 after DES treatment. In the C 1s spectra (Fig. 5B), the peak corresponding to C–O bonds at 286.3 eV decreased in DLNP compared to KL. The increase in the C/O ratio and the reduction in C–O bonds in DLNP indicate the cleavage of ether bonds in KL and the occurrence of C–C bond condensation during depolymerization. Additionally, in the O 1s spectra (Fig. 5C), an increase in the proportion of phenolic oxygen and a decrease in the proportion of C=O bonds were observed in DLNP. This is attributed to the removal of cellulose and hemicellulose during the depolymerization process. Furthermore, the removal of these carbohydrates may have contributed to the increase in the C/O ratio and the reduction in C-O bond content in DLNP.

**Fig. 6:**
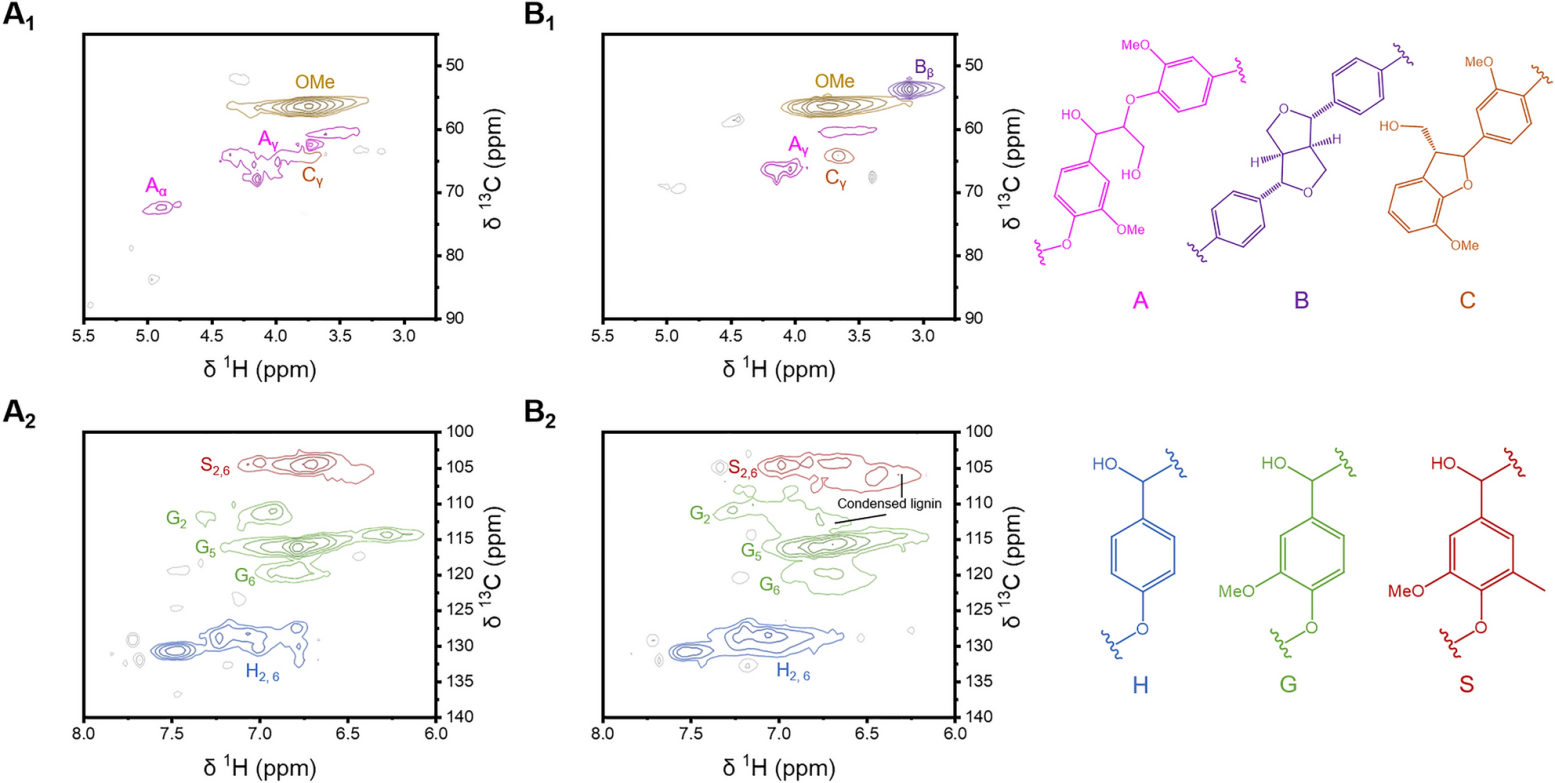
2D–HSQC NMR spectra of (**A**) KL and (**B**) DLNP, showing (1) side-chain regions and (2) aromatic regions.

The 2D–HSQC NMR technique enables the observation of interactions between heteronuclei, allowing for the identification of specific lignin bonding patterns that cannot be determined through FTIR, ^31^P-NMR, and XPS. To analyze changes in major linkages and structural units before and after depolymerization using DES, the side-chain region (δ_C_/δ_H_ 45.0–90.0/2.5–5.5 ppm) and aromatic region (δ_C_/δ_H_ 100.0–140.0/6.0–8.0 ppm) of the 2D–HSQC NMR spectra of KL and DLNPs were compared, as shown in Fig. 5. The assignment of key lignin signals in the spectra was based on previous literature^49–51^. In the side-chain region (Fig. 6A1 and B1), KL exhibited peaks corresponding to the methoxyl group (55.0–60.0/4.2–3.3 ppm) and β-O-4 linkages (A_γ_: 60–70/3.3–4.5 ppm, A_α_: 69.0/4.9 ppm). In contrast, the A’_γ_ signal in DLNP was weakened, and the A_α_ signal disappeared completely, indicating that the β-O-4 linkages were cleaved during depolymerization with DES. Furthermore, the phenylcoumaran structure (C_γ_, 64.0/3.7 ppm), which was weakly present in KL, showed slight strengthening in DLNP. New signals corresponding to the pinoresinol structure (${\text{B}}_{\beta }$: 50.7–52.6/3.2–2.7 ppm) appeared in the side-chain region of the DLNP spectrum, with strong intensity^47,52,53^. The enhancement of these signals provides evidence of condensation reactions that occurred during the depolymerization process.

In the aromatic region of the 2D–HSQC NMR spectra (Fig. 6A2 and B2), signals corresponding to H, G, and S units in lignin were clearly observed in both KL and DLNPs (H: 126.0–134.0/6.7–7.7 ppm, G: 110.0–121.3/6.1–7.4 ppm, S: 102.5–107.4/6.3–7.1 ppm). However, in contrast to KL, the signals for lignin units in DLNP appeared over a much broader range, indicating a variety of chemical shifts resulting from the depolymerization process, including condensation reactions^54,55^. These 2D–HSQC NMR results highlight the significant impact of the depolymerization process on the chemical properties and bonding patterns of lignin.

**Fig. 7:**
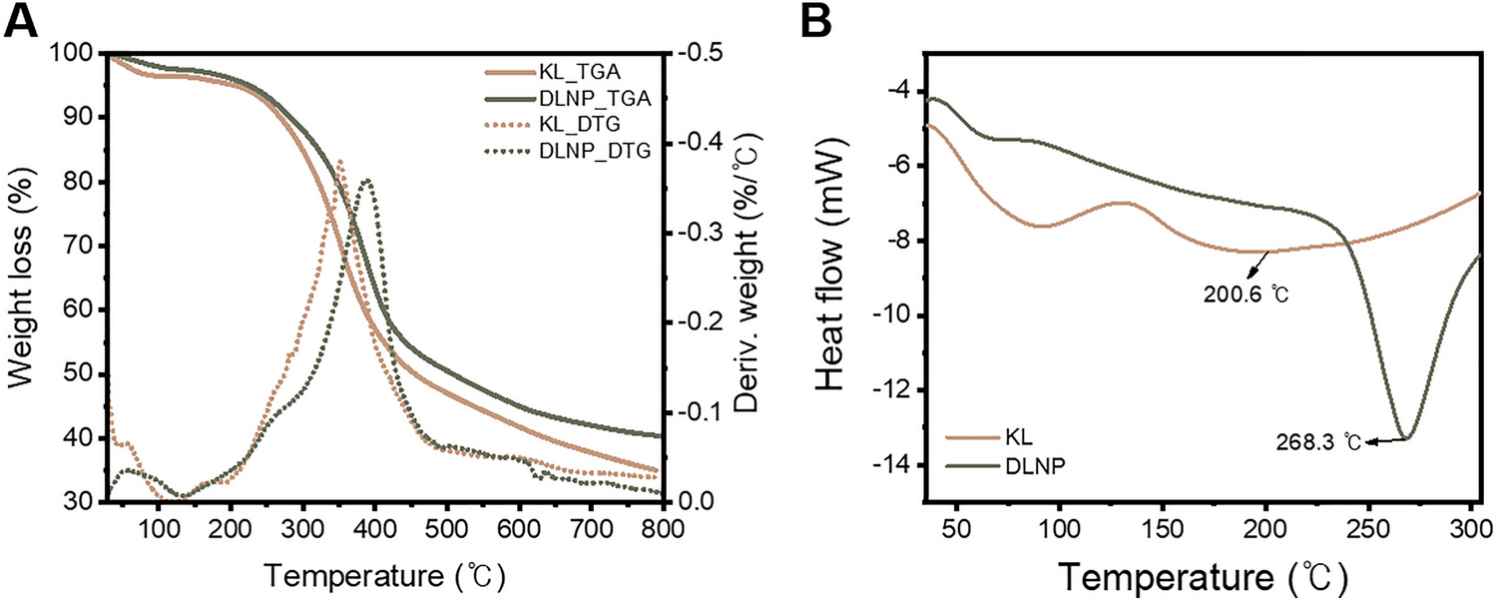
(**A**) TGA, DTG, and (**B**) DSC curves of KL and DLNP.

In general, the aim of lignin depolymerization is to produce monomers, so the occurrence of condensation reactions, which are a side effect of depolymerization, is typically suppressed as much as possible during the depolymerization process^56–58^. However, in the context of lignin fractionation for utilizing macromolecules, condensation can confer beneficial properties. The cleavage of ether bonds and the formation of C–C bonds during condensation are known to enhance the thermal stability of lignin^59^. From this perspective, TGA and DSC analyses were performed to evaluate the thermal properties of DLNPs (Fig. 7, Table 3). The TGA and DTG curves of KL and DLNPs (Fig. 7A) reveal decomposition occurring between 60 and 800 °C. Weight loss between 60 and 140 °C corresponds to the removal of moisture and low-molecular-weight volatile substances caused by initial heating of the sample. Then, starting at 150 °C, lignin undergoes its first pyrolysis stage, where ether bonds decompose, releasing phenols, alcohols, and methoxyl groups, along with the degradation of aliphatic side chains in the 200–300 °C range. KL exhibits an onset temperature (T_onset_) of 206.4 °C, whereas DLNP shows a higher T_onset_ of 226.0 °C. Additionally, DLNP shows less weight loss than KL in this region. This reduction can be attributed to the cleavage of ether bonds, particularly β-O-4 bonds, during the depolymerization process. The second pyrolysis stage, occurring between 300 and 400 °C, marks the decomposition of lignin’s main skeletal structure, including C–C bonds. In this range, KL has a maximum decomposition temperature (T_max_) of 352.0 °C, while DLNP exhibits a T_max_ of 388.5 °C, a significant increase of 36.5 °C. Lignin with a higher content of condensed phenols typically shows increased T_max_ values^60^, as seen in DLNP, which indicates the presence of condensed structures formed during DES-based depolymerization. Additionally, these condensed C–C bond structures in DLNP contribute to the carbonization of lignin, resulting in relatively higher char residue compared to KL after finishing the decomposition and condensation of lignin’s aromatic structure above 400 °C. In summary, TGA results demonstrate that DLNP exhibits improved thermal stability due to the formation of these condensed structures during the depolymerization process using DES.

**Table 3:** Thermal parameters of KL and DLNP analyzed by TGA and DSC.

	TGA			DSC	
	T_onset_ (°C)	T_max_ (°C)	Char (%)	Endothermic temperature (°C)	Enthalpy (J/g)
KL	206.4	352.0	35.0	200.6	193.8
DLNP	226.0	388.5	40.4	268.3	268.4

The results of the DSC analysis for KL and DLNP are presented in Fig. 7B and Table 3. The DSC curves for both KL and DLNP display endothermic peaks in the range of 60–140 °C, corresponding to the removal of moisture and low-molecular-weight volatile substances. The higher endothermic enthalpy of KL in this range aligns with the greater weight loss observed in the TGA curves. Above 150 °C, where the thermal degradation of lignin begins, the DSC curve for KL shows a broad endothermic peak, whereas the curve for DLNP features a sharp and narrow peak above 225 °C. This difference in peak shape arises from structural changes in lignin during the depolymerization process. While KL retains its ether bonds, DLNP undergoes significant cleavage of β-O-4 bonds. Additionally, the endothermic enthalpy for DLNP is significantly higher at 268.4 J/g compared to 193.8 J/g for KL, indicating that more energy is needed to break the bonds in DLNP due to the stable condensed structures formed within the lignin^61^. These TGA and DSC results confirm that the thermal stability of DLNP is enhanced by the condensed structures produced during the depolymerization process using DES. These properties suggest that DLNP has potential applications in fields such as flame retardants, composite, and carbon materials.

## Conclusion

This study presents an eco-friendly approach to lignin depolymerization and the production of LNPs using DES. The use of DES effectively facilitated lignin depolymerization, resulting in a lower molecular weight and enhanced homogeneity. The DLNP produced through this continuous process exhibited a high negative surface charge, contributing to uniform particle size and long-term stability in dispersion. Analyses via FTIR, ^31^P-NMR, and 2D–HSQC NMR indicated that lignin depolymerization primarily involved the cleavage of β-O-4 bonds while preserving the aromatic structure, with the formation of condensed structures such as pinoresinol and phenylcoumaran during the depolymerization process. Moreover, the thermal analysis demonstrated that DLNP displayed superior thermal properties compared to KL, attributed to the presence of these condensed structures, suggesting its potential for use in high-temperature applications. This study highlights that DES-based lignin depolymerization and LNP production is an ecofriendly and efficient alternative to traditional organic solvent–based methods, paving the way for high-value applications of lignin-based biomaterials.

## Data Availability

The data that support the findings of this study are available from the corresponding author, (Hyo Won Kwak), upon reasonable request.
